# Neurophysiological correlates of decision-making efficiency in depressive disorders

**DOI:** 10.1192/j.eurpsy.2025.1332

**Published:** 2025-08-26

**Authors:** A. F. Iznak, ?. V. Iznak, A. F. Beresneva

**Affiliations:** 1 Laboratory of Neurophysiology, Mental Health Research Centre, Moscow, Russian Federation

## Abstract

**Introduction:**

Reduced ability to make adequate decisions significantly affects the daily functioning and social adaptation in depressive patients. Neurophysiological bases of reduced efficiency of decision-making based on both logical reasoning and emotional processes are not sufficiently studied.

**Objectives:**

The aim of the study was to search for possible links between signs of decision-making efficiency and EEG parameters in depressive patients.

**Methods:**

The study involved 48 depressive female in-patients (F31.3-4, F34.0, by ICD-10), aged 16-25 years (mean age 18.4 ± 2.6). To assess the decision-making process, computer versions of the Wisconsin Card Sorting Test (WCST) and the Iowa Game Task (IGT) were used. Multichannel resting EEG recordings with spectral power analysis were performed at the same visit before the start of treatment. The rank correlation analysis (Spearman) was used for statistical data processing.

**Results:**

In WCST test, negative correlations (p<0.05) were found between the “percentage of conceptual level responses”, and the spectral power values of beta1 (13-20 Hz), beta2 (20-30 Hz) in the central and posterior areas, and theta1 (4-6 Hz) in anterior areas of the cerebral cortex. Delta (2-4 Hz) activity values in the left frontal areas positively correlated with the WCST parameters “number of moves spent on completing the first category”, “number of perseverations on the previous move”, “number of moves” and “test completion time”. Thus, patients complete the task longer and less effectively with frontal hypoactivity and increased activation of the posterior areas of the cortex that reflects a violation of the brain’s inhibitory mechanisms. In the IGT test, positive correlations (p<0.01) were revealed between the number of choices of the “bad” deck (A) and both “bad” decks (A and B), as well as negative correlations between the preference for “good” (C and D) over “bad” (A and B) decks in the second half of testing and the values of the alpha2 (9-11 Hz) spectral power in the anterior areas of the left hemisphere. The preference for “good” over “bad” decks on moves 40–60 negatively correlated with the values of alpha3 (11-13 Hz) and beta2 (20-30 Hz) in the right frontal and central areas. Thus, decision-making deficit in the IGT was associated with a relative decrease in the activation of the anterior regions of the left hemisphere and hyperactivation of the right hemisphere.

**Conclusions:**

In depressive patients, impairments in the logical reasoning task (WCST) are associated with hypofrontality while impairments in the emotional learning task (IGT) − with disbalance of hemispheric activation, namely with hypoactivation of the frontal regions of the left hemisphere and increased activation of the right hemisphere.

**Disclosure of Interest:**

None Declared

